# Dynamic profiling of double-stranded RNA binding proteins

**DOI:** 10.1093/nar/gkv726

**Published:** 2015-07-16

**Authors:** Xinlei Wang, Lela Vukovic, Hye Ran Koh, Klaus Schulten, Sua Myong

**Affiliations:** 1 Department of Bioengineering, University of Illinois at Urbana-Champaign, Urbana, IL 61801, USA; 2 Center for the Physics of Living Cells, University of Illinois at Urbana-Champaign, Urbana, IL 61801, USA; 3 Institute for Genomic Biology, University of Illinois, 1206 W. Gregory St,. Urbana, IL 61801, USA; 4 Department of Physics, University of Illinois at Urbana-Champaign, Urbana, IL 61801, USA; 5 Biophysics and Computational Biology, University of Illinois, 1110 W. Green St., Urbana, IL 61801, USA

## Abstract

Double-stranded (ds) RNA is a key player in numerous biological activities in cells, including RNA interference, anti-viral immunity and mRNA transport. The class of proteins responsible for recognizing dsRNA is termed double-stranded RNA binding proteins (dsRBP). However, little is known about the molecular mechanisms underlying the interaction between dsRBPs and dsRNA. Here we examined four human dsRBPs, ADAD2, TRBP, Staufen 1 and ADAR1 on six dsRNA substrates that vary in length and secondary structure. We combined single molecule pull-down (SiMPull), single molecule protein-induced fluorescence enhancement (smPIFE) and molecular dynamics (MD) simulations to investigate the dsRNA-dsRBP interactions. Our results demonstrate that despite the highly conserved dsRNA binding domains, the dsRBPs exhibit diverse substrate specificities and dynamic properties when in contact with different RNA substrates. While TRBP and ADAR1 have a preference for binding simple duplex RNA, ADAD2 and Staufen1 display higher affinity to highly structured RNA substrates. Upon interaction with RNA substrates, TRBP and Staufen1 exhibit dynamic sliding whereas two deaminases ADAR1 and ADAD2 mostly remain immobile when bound. MD simulations provide a detailed atomic interaction map that is largely consistent with the affinity differences observed experimentally. Collectively, our study highlights the diverse nature of substrate specificity and mobility exhibited by dsRBPs that may be critical for their cellular function.

## INTRODUCTION

While all cellular RNA molecules are synthesized in single-stranded (ss) form, many can form into secondary structures that encompass segments of double stranded (ds) RNA. Hence, dsRNA molecules are common in cells and are recognized as critical regulatory factors in many biological processes (1–3). For example, dsRNA regions are present in the precursors of microRNAs, siRNAs, messenger RNA (mRNA), transfer RNA (tRNA), as well as in the genome of RNA viruses that can be released into cells upon infection.

The family of proteins responsible for processing dsRNA is called double stranded RNA binding proteins (dsRBP). Various dsRNAs serve as cargoes, activators and substrates of dsRBPs in many biological pathways (4,5). For example, certain dsRNA structures found in viruses activates protein kinase R (PKR), which in turn triggers the downstream antiviral immune pathways (6,7); pri-microRNAs are recognized and cleaved by Drosha-DGCR8 to produce pre-microRNA in the nucleus; pre-microRNA is cleaved by Dicer-TRBP to form into mature microRNA (8,9).

The dsRBP family is defined by the presence of one or more double-stranded RNA binding domains (dsRBD) (10). The dsRBDs are highly conserved in amino acid composition and domain structures and are found across various species (11–13). Despite the high degree of conservation, dsRBPs are involved in diverse biological functions where they interact with variety of RNA substrates. The RNA substrates vary in secondary structure and differ in length of duplex. While the biological functions of dsRBPs are known, it remains uncertain if dsRBPs exhibit certain substrate specificity.

Two types of dsRBDs are found in dsRBPs; type-1 dsRBD (dsRBD-I) usually binds dsRNA while type-2 (dsRBD-II) is mainly involved in protein–protein interaction (10,14). The number of dsRBD-I present in each dsRBP is highly variable (5); for instance, ADAD2 contains only one, whereas ADAR1 contains three dsRBD-Is. It is currently unknown why some dsRBPs need multiple units while others possess a single dsRBD-I, and if the number of the dsRBD-I is correlated with the protein's affinity to dsRNA. dsRBD-I adopts an ‘α-β-β-β-α’ structure, which contacts dsRNA in three grooves (minor–major–minor) along a stem spanning 15 base pairs (bp) (11,12). This protein–RNA binding mode is structure- but not sequence-dependent since dsRBDs recognize the A-form helical axis of dsRNA rather than the specific RNA sequence (4,15). While dsRBD-I of ADAR2 recognizes and binds dsRNA at certain mismatch locations (16), it is not clear to what extent other dsRBDs contribute to binding dsRNA and highly structured dsRNA.

To address some of these outstanding questions, we examined dsRNA interaction with four dsRBPs: ADAD2, TRBP, Staufen1 and ADAR1. We chose dsRBPs that contain different number of dsRBD-I units and participate in various cellular functions. ADAD2 has only a single dsRBD-I, followed by Staufen1 and TRBP containing two, and ADAR1 possessing three units of dsRBD-Is. In terms of biological function, both ADAD2 and ADAR1 are RNA deaminases that edit adenosine to inosine (A to I) in mRNA and microRNA precursors (17). Staufen1 is responsible for mRNA transport to dendrites in neurons where its tubulin binding domain likely binds microtubules along axons (18). In addition, human Staufen1 binds to the 19bp stem in ARF1 mRNA and a to intermolecular Alu element-Alu element duplexes for the purpose of Staufen-mediated mRNA decay (19,20) and intramolecular Alu element-Alu element duplexes to compete with mRNA retention in paraspeckles (21). TRBP is a key player in the RNA Induced Silencing Complex (RISC) assembly (22) and also modulates the initiation of HIV-1 gene expression (23,24).

We investigated the binding affinity of the four dsRBPs toward six different dsRNA substrates with varying length and secondary structure. The length variants include 25, 40 and 55 bp dsRNA whereas the imperfectly base-paired/structured RNA includes pre-let7 (pre-microRNA), TAR RNA and tRNA-like RNA. All proteins were overexpressed in mammalian cells (HEK 293) and pulled down to a single molecule imaging surface coated with the appropriate antibody (25). Fluorescence-labeled RNA substrates were added to test their binding affinity. We report on the dynamics motion involved in some protein–RNA interaction pairs probed by single molecule Protein Induced Fluorescence Enhancement (smPIFE) (26). In addition, we examined the molecular interface of the RNA interacting domains of dsRBPs through MD simulations. Our study reveals that despite the presence of highly conserved dsRNA binding domains, the dsRBPs tested on our assay platform display substantial differences in their substrate specificity and degree of dynamics on RNA substrates.

## MATERIALS AND METHODS

### RNA labeling and annealing

The sequences of all RNA substrates are displayed in Supplementary Table S1. Pre-let7, TAR and tRNA molecules were purchased from IDT as single strand RNA with fluorescent label at the 5′ end. 25, 40 and 55 bp dsRNAs were purchased from Dharmacon as separate single strand RNA and 3′-DY547 was incorporated in the process of each RNA synthesis. For dsRNA annealing, two complementary RNA strands were mixed in equal concentration in annealing buffer (100 mM NaCl and 10 mM Tris at pH 8) and heated at ∼90°C for 2 min and gradually cooled to room temperature. U40 with 3′ amine modification was purchased from IDT and labeled with Cy3 NHS ester dye from GE Healthcare (27). Briefly, the dye was mixed at 2-fold molar excess concentration with RNA containing 3′ amine modifier, in a buffer containing 100 mM NaHCO_3_ at pH 8.5 and then incubated overnight. Unreacted dye was removed by two rounds of ethanol precipitation. The resulting labeling efficiency was ∼90%. For tRNA preparation, we incubated 1 μM of tRNA in a buffer containing 10 mM Tris, 50 mM NaCl, 3 mM MgCl_2_, pH 7.5 at 90°C for 2 min and quickly cooled it down on ice. Due to the protocol, we expect the tRNA sample to include some misfolded, yet secondary structured RNAs (28). Therefore, we refer to this RNA mixture as tRNA-like RNA.

### Protein lysate preparation

ADAD2, TRBP and Staufen1 were cloned from Human Open Reading Frame Library and a C-terminal EYFP was added to each protein sequence. Then, C-terminal EYFP-TRBP, C-terminal EYGP-Staufen1 and N-terminal EGFP-ADAR1 were overexpressed in human A549 cells, and C-terminal EYFP-ADAD2 was overexpressed in HEK293 cells. Cells were lysed using RIPA (Thermo Scientific RIPA Lysis and Extraction Buffer, Catalog number: 89900) 24 h after transfection and cell lysates were collected and centrifuged; finally supernatants were collected for each protein. The dsRBP levels were quantified by fluorometry measurement based on EYFP or EGFP intensity using cy5 dye as standard (see Supplementary Figure S2A, B). Cell lysates were stored in −80°C for later use.

### Single molecule pull down assay (SiMPull)

Polyethylene glycol (PEG)-coated quartz slides with flow chambers were obtained according to previously published protocol (29). A PEG surface was coated with Neutravidin (0.05 mg/ml) followed by anti-GFP (RABBIT, 5 μg/ml) antibody conjugated with biotin (Rockland 600-406-215) and incubated for another 5min in T50 (10 mM Tris pH 8 and 50 mM NaCl). About 400 pM of C-terminal EYFP or N-terminal EGFP fused dsRBP cell lysates were added to the antibody coated surface and incubated for 5–10 min.

To measure the level of dsRBPs on the PEG surface, we used the level of TRBP-EYFP as standard for all dsRBPs, since the concentration of four dsRBPs were calibrated and dilution factors were normalized to make sure they were applied at the same level on the PEG surface. To measure the level of TRBP after SM Pull-down using biotinylated GFP antibody, we used anti-TRBP antibody (Abcam [1D9](ab129325)) and Anti-mouse Alexa Fluor^®^ 488 Conjugate (Cell Signaling #4408). We note that anti-GFP antibody is sufficient to pull down EYFP as well as GFP.

### Protein induced fluorescence enhancement (PIFE) assay

Single-molecule detection of protein–RNA interaction dynamics assay was achieved employing custom-built prism-type total internal reflection fluorescence (TIRF) microscopy (30,31). After pulling down dsRBP using GFP antibody, 1nM of fluorescently labeled RNA substrate was added and incubated for 5min. During data acquisition, an oxygen scavenging buffer (0.5% (wt/vol) glucose, 10 μg/μl glucose-oxidase (Sigma) and 8.8 kU/ml catalase (Calbiochem)) was used with 2.5 mg/ml trolox (Sigma) to stabilize fluorophore and together with 20 mM Tris pH7.5 and 25 mM NaCl in imaging buffer system for all the protein–RNA interaction. The exposure time was 30 ms and the single molecule signals were processed with a custom-edited IDL and Matlab program.

### Structural models of RNA constructs and dsRBDs

In order to complement the experimental studies, we examined structural models of RNA constructs used in experiments and several of the dsRNA binding domains (dsRBDs). Structures of U40 and 25, 40 and 55 bp dsRNA were prepared with the software 3DNA (32). 3D structures of TAR RNA and pre-let7, shown in Supplementary Figure S1, were prepared with the software 3dRNA (33), based on the lowest free energy secondary structure predictions obtained from the RNA structure web server (34).

To compare dsRNA-binding interfaces of studied dsRBDs, we either examined existing crystal structures or prepared homology models as described below. dsRBDs with known structures include TRBP dsRBD 1 (pdbID 3LLH), TRBP dsRBD 2 (pdbID 3ADL), and ADAR1 dsRBD 3 (pdbID 2MDR). The homology models of three additional dsRBDs were prepared employing the Protein Model Portal (35): Staufen1 dsRBDs 2 (based on pdbID 1STU; 61% sequence identity), Staufen1 dsRBD3 (based on pdbID 1UHZ; 78% sequence identity), and ADAD2 dsRBD (model based on pdbID 1x47; sequence identity 32%). Despite some of the models and templates having low sequence identities, all the prepared models had their conserved dsRNA-binding residues located on one surface of the domain, forming three distinct regions that can bind successive minor–major–minor grooves of the RNA duplex, as is usually observed for dsRBDs (28).

The stability of prepared homology models of dsRBDs was examined through PACE hybrid resolution model simulations, where dsRBDs were described with a united-atom model, placed in a coarse-grained solvent model (36,37). PACE molecular dynamics was validated through simulations of TRBP dsRBD 2, whose secondary and tertiary structures remained stable in 80 ns simulations. After quick and slight initial readjustment of tertiary structure, ADAD2 dsRBD and Staufen1 dsRBD 2 remained stable in 160 and 167 ns simulations, respectively, with their dsRNA binding residues remaining aligned along one of the domain surfaces (which enables binding to dsRNA). In simulations of Staufen1 dsRBD 3, the major groove KKxxK motif and the loop that usually binds the minor groove remained stable, while the N-terminal helix did not find its equilibrium conformation in 270 ns of simulation.

To examine the binding strength between dsRNA and individual dsRBDs, we simulated complexes of dsRNA with: (a) TRBP-dsRBD2, based on pdbID 3ADL, (b) TRBP-dsRBD1, based on pdbID 3LLH, (c) ADAR1-dsRBD3, based on pdbID 2MDR, (d) ADAD2 dsRBD, based on the above described homology model. Initial structures of complexes were based on the crystal structure of TRBP-dsRBD2 bound to coaxially stacked RNA duplexes (pdbID 3ADL), where the resolved RNA was replaced by a 35-bp RNA duplex, prepared with the software 3DNA (30). The system preparation for all complexes was performed as described for TRBP-dsRBD2: dsRNA in (28). MD simulations were performed with the program NAMD2 (38), employing the AMBER force field with SB and BSC0 corrections (39,40). The particle-mesh Ewald (PME) method (41) was used for evaluation of long-range Coulomb interactions. The time step was set to 1.0 fs, for dsRNA in complex with TRBP-dsRBD1 and TRBP-dsRBD2, or 2 fs, for dsRNA in complex with ADAR1-dsRBD3 and ADAD2 dsRBD. Long-range interactions were evaluated every 2 fs (van der Waals) and 4 fs (Coulombic). After 2000 steps of minimization, ions and water molecules were equilibrated for 2 ns around complexes, which were constrained using harmonic forces with a spring constant of 1 kcal/(mol Å^2^). Then, unconstrained complexes were simulated for 100 ns (TRBP dsRBDs) or 55 ns (ADAR1, ADAD2 dsRBDs). The simulations were performed in NpT ensemble, at a constant temperature *T* = 310 K, a Langevin constant *γ*Lang = 1.0 ps^−1^, and at a constant pressure *p* = 1 bar.

## RESULTS

### Single molecule pull-down of dsRBPs

**Figure 1.**
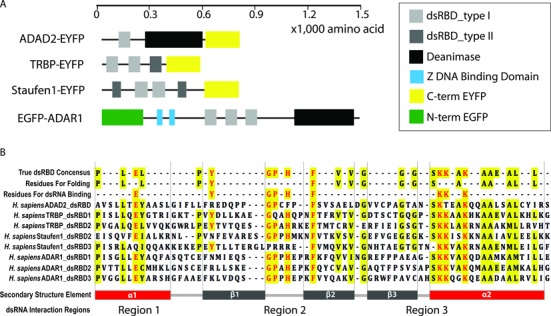
Double stranded RNA binding proteins (dsRBPs). (**A**) Functional domains of four dsRBPs. ADAD2, TRBP2 and Stau1 were fused with EYFP at the C-terminus, while ADAR1 was fused with GFP at the N-terminus. (**B**) Amino acid sequence information of the type-1 dsRBDs in ADAD2, TRBP2, Stau1 and ADAR1. The top line shows the consensus residues of dsRBDs, which are highlighted in yellow. The second line shows the key residues critical for folding into the dsRBD conserved αβββα structure. The third line shows the residues necessary for dsRNA binding, highlighted in red. Secondary structure α-β-β-β-α sections are labeled along with dsRNA binding regions, 1, 2 and 3.

Four dsRBPs, ADAD2, TRBP, Staufen1 and ADAR1 were chosen because they all have at least one dsRBD-I (type I dsRBD) with highly conserved amino acid residues (Figure 1A and B). However, the dsRBPs differ in total length (600–1500 amino acids) as well as in overall domain composition. ADAD2 and ADAR1 possess a large deaminase domain whereas TRBP and Staufen1 encompass dsRBD-II (type II dsRBD) domains (Figure 1A). We sought to set up a single molecule affinity testing platform in which one can immobilize the same number/density of dsRBP and apply varying RNA substrates. The single molecule pull-down assay (25) was implemented to isolate dsRBPs directly from mammalian cells. The full length of each dsRBP fused with an EYFP or EGFP tag was over-expressed in HEK293 cells (Figure 1A). The cell lysate was obtained and the intensity of EYFP/EGFP was measured to test the dsRBP expression level. We note that EYFP and EGFP constructs used here are in the monomeric form which should not dimerize (42).

**Figure 2.**
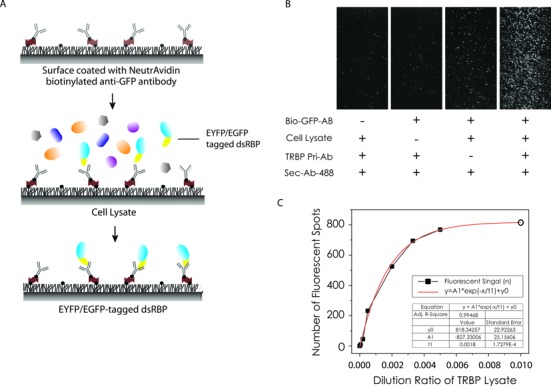
Single molecule pull down (SiMPull) of dsRBPs. (**A**) Schematic for single molecule pull down of dsRBP from cell lysate using single molecule imaging surface treated with antibody against EYFP/GFP. (**B**) TIRF images for TRBP pulled down from cell expressing TRBP-EYFP and controls by omitting the indicated item one at a time. (**C**) The number of TRBP proteins from the serially diluted TRBP cell lysate using primary antibody against TRBP and corresponding fluorescent secondary antibody.

Upon determining the concentration of each protein based on the fluorescence intensity of EYFP/EGFP (Supplementary Figure S2A and B), the same concentration of each dsRBP was applied on a single molecule surface coated with anti-GFP/anti-YFP antibody (24) (Figure 2A). The specificity of dsRBP binding to the surface was confirmed by adding serially diluted cell lysate to anti-GFP coated surface and applying primary and fluorescence (A488) labeled secondary antibody for the corresponding dsRBP (Figure 2B). We confirmed the dsRBP binding specificity and obtained an accurate count of TRBP molecules on the surface (Figure 2B). The number of countable TRBP molecules on the surface was saturated as the concentration of cell lysate increased (Figure 2C).

### Relative binding affinity of dsRBPs to various RNAs

**Figure 3.**
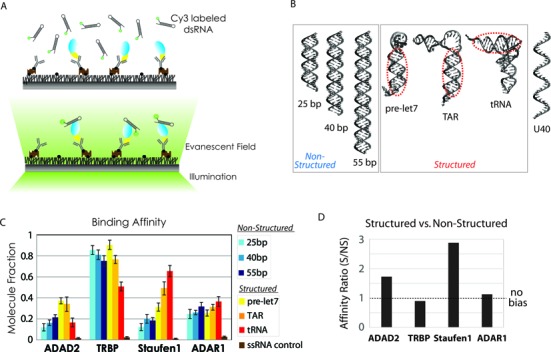
Relative binding affinity of dsRBPs to various dsRNAs. (**A**) Schematic illustration of fluorescence labeled dsRNA bound by dsRBP on the PEG-coated surface. 1 nM dsRNAs were used in all cases. (**B**) Secondary structure of six dsRNAs tested and the ssRNA of U40 tested as a negative control. Regions marked by red dotted circles involve a set of minor–major–minor grooves, which suffice for one dsRBD interaction with dsRNA. (**C**) The relative binding affinity of each dsRBP to six dsRNAs shown in Figure 3B. The fraction was calculated as the ratio of the number of dsRNA molecule measured by cy3 detection and the number of dsRBP molecules detected by immuno-fluorescence measurement against EYFP/GFP at single molecule level. (**D**) dsRBP preference to structured or non-structured dsRNA in terms of relative binding affinity. The categories of structured and non-structured RNA are shown in (B).

To investigate the RNA binding specificity of the four dsRBPs, 1 nM of Cy3-labeled RNA molecules was applied to the dsRBP-immobilized imaging surface (Figure 3A). The fluorescence of EYFP or EGFP on dsRBPs does not interfere with the detection of Cy3 signal due to the extremely fast photobleaching of both fluorescent proteins. The RNA substrates that differ in duplex length and secondary structure were prepared. The length variants, 25, 40 and 55 bp dsRNA were categorized as ‘non-structured’ and the structure variants, pre-let7, TAR and tRNA-like RNA as ‘structured’ RNA (Figure 3B). We termed the last RNA ‘tRNA-like RNA’ because our tRNA preparation involves heating at 90°C for 2 min followed by a rapid cooling, which should result in mixed population of properly folded tRNA along with misfolded, yet secondary structured RNA (28). We refer to this RNA mix as ‘tRNA’ for the remainder of the paper. We confirmed that this RNA preparation does not lead to aggregation. If the RNA aggregated, we would see multi-step photobleaching in our single molecule detection platform because every molecule of RNA is fluorescently labeled. The single step photobleaching that we detect in majority of molecules indicate that RNA exist as monomers. We note that all structured RNA still retained a long dsRNA stem sufficient for dsRBP binding (Supplementary Figure S1). The single strand (ss) RNA composed of 40-uracil (U40) was included as a negative control.

After checking the density of dsRBP molecules on the imaging surface, we applied Cy3 labeled RNA, washed out the unbound RNA and visualized the fluorescence signal by home-built total internal reflection fluorescence (TIRF) microscope (Figure 3A). The single molecule images from multiple areas were taken to count the number of dsRNA bound to proteins on the surface. For each RNA, the average number of RNA bound per field of view was used as a proxy for the comparative binding affinity of the corresponding RNA–dsRBP pair. The nonspecific binding of dsRNA was checked by omitting cell lysates with overexpression of individual dsRBPs and by applying a negative control, U40 ssRNA. Both showed negligible binding, suggesting that the fluorescence signals arise from the specific binding of dsRNA to dsRBP molecules (Supplementary Figure S3A).

We quantified the fraction of RNA-bound dsRBP to total dsRBP molecules (sum of bound- and unbound molecules) for each dsRBP with six dsRNAs and one ssRNA. Amongst the four dsRBPs, TRBP showed the highest relative binding affinity to all substrates, mostly ranging between 80 and 90%, except for the reduced binding to tRNA (∼50%). Staufen1, ADAR1 and ADAD2 exhibit substantially lower affinities to all RNAs in general; on average, 20–40% of protein was occupied by RNA (Figure 3C). Notably, the comparative binding affinities of dsRBPs toward RNA are not correlated with the number of dsRBD-I. For instance, ADAR1 with three dsRBD-Is and ADAD2 with only one dsRBD-I displayed similar binding affinities for RNA substrates whereas TRBP with two dsRBD-Is exhibited the highest affinity. To test if TRBP alone is primarily responsible for binding dsRNA, we performed EMSA where we subjected three Cy3 labeled dsRNA substrates to both TRBP overexpressed cell lysate and purified TRBP. The result shows that TRBP–RNA complex from both are comparable (Supplementary Figure S2C and D). This also confirms that the EYFP tagging does not interfere with dsRNA binding (43). Together, our data indicates that the number of type-1 dsRBDs may not be a major factor for determining dsRBP-dsRNA affinity. In agreement with previous report (44,45), Staufen1 interacts with itself at high concentration range (>100 nM) as shown by EMSA assay (Supplementary Figure S2E).

The four dsRBPs also displayed different binding affinities to various structural features of RNAs. To compare the binding propensity toward structured RNA, we obtained the average of all bound fractions corresponding to structured (pre-let7, TAR and tRNA) and non-structured RNA (25, 40 and 55 bp) substrates for each dsRBP (Figure 3C) and calculated the ratio between structured versus non-structured (S/N), termed here affinity ratio (Figure 3D); ratio of 1 indicates no bias to either type whereas a ratio >1 reflects a preference toward structured RNAs. Interestingly, ADAD2 and Staufen1 showed significantly higher affinity for the structured RNAs with the affinity ratio approaching 2:3 (Figure 3D). On the other hand, TRBP and ADAR1 have similar affinities for almost all the RNA substrates (Figure 2D). Unexpectedly, all the proteins displayed substantial affinity to highly structured tRNA-like RNA, which can be in part due to tRNA which has a complex L-shape with only a short dsRNA portion of 13–14 bp and the mixed population of misfolded RNA. (46)To further test the binding of tRNA, we performed two types of competitive binding assays. First, we applied equimolar concentration of Cy3 labeled tRNA and Cy5 labeled 27bp dsRNA to single molecule surface coated with individual dsRBPs. We observed that the binding affinity and preference for tRNA versus dsRNA exhibited the same pattern as our previous assay shown in Figure 3C (Supplementary Figure S3B). This result confirms that tRNA binds to dsRBPs, albeit to varying degrees and that Staufen1's binding preference for structured RNA is retained even in the presence of dsRNA. Second, we added tRNA to dsRBPs pre-bound with dsRNA and observed that tRNA still exhibited sufficient level of competitive binding (Supplementary Figure S3C). Furthermore, dsRBD–tRNA binding model is plausible: in one possible binding mode, dsRBD2 of TRBP fits in the duplex-like region of tRNA, and the contact area between dsRBD and tRNA is comparable to values observed for regular dsRNA, even slightly higher, as the ssRNA tail of tRNA can also bind to the dsRBD on the side (Supplementary Figure S3D).

In conclusion, the four tested dsRBPs exhibit different substrate specificities in our single molecule platform, which may arise from different binding affinities toward RNA substrates with varying secondary structures. Furthermore, RNA binding affinity of dsRBP does not seem to depend on the number of its dsRBD-I.

### Dynamic property of dsRBP-dsRNA interaction

Previously, we had reported that TRBP exhibits ATP-independent sliding/diffusion activity along dsRNA (47). The motion of TRBP on dsRNA, detected by single molecule FRET (Förster Resonance Energy Transfer) exhibited repetitive movement from one end to the other end of a dsRNA strand (48). When TRBP forms a complex with Dicer, such movement induced by TRBP serves to accelerate RNA cleavage. Two orthologous proteins, PACT and R3D1-L, also displayed the same sliding activity on dsRNA, suggesting a possibility that such mobility can be conserved among dsRBPs. Here, we examined if the same sliding dynamics can be observed in the dsRBP-RNA interaction.

**Figure 4.**
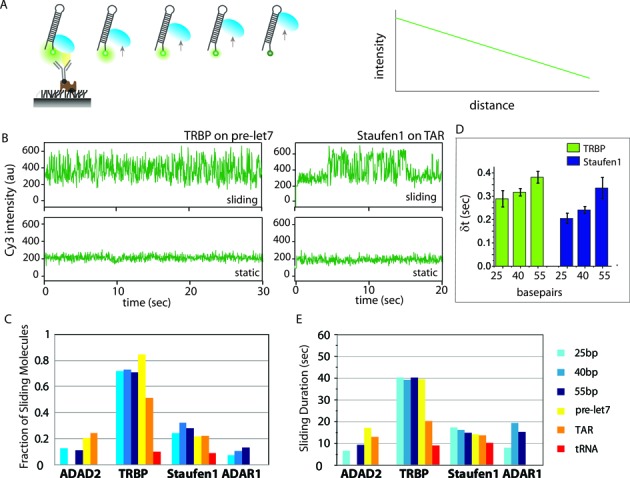
Dynamics property of dsRBP–dsRNA interaction. (**A**) Schematic of single molecule protein induced fluorescent enhancement (smPIFE) (left). Fluorescence intensity is dependent on the distance between protein and dye. (**B**) Two different types of smPIFE traces observed in two dsRBP–dsRNA interaction cases. The top two panels are the traces of sliding and static molecules in TRBP2-Prelet7 interaction, respectively. The bottom two panels are the traces of sliding and static molecules in Stau1–TAR RNA interaction, respectively. (**C**) Quantification of the percentage of protein molecules showing sliding behavior along an RNA duplex for each dsRBP–dsRNA interaction. The percentage was calculated as the ratio of protein molecules showing sliding behavior out of the total of protein molecules bound to dsRNA. For each dsRBP–dsRNA interaction, >300 traces were examined to calculate the percentage. (**D**) Dwell time analysis of peak to peak interval from smPIFE trace. It is the average time for protein to complete one round of sliding along dsRNA axis. (**E**) Quantification of sliding time duration averaged over 50–100 traces exhibiting sliding activities for each dsRBP–dsRNA interaction case.

The sliding activity of dsRBPs was detected using single molecule protein induced fluorescent enhancement (smPIFE) (26), in which the change in distance between protein and fluorescent dye is indicated by the intensity change of the dye (31). Briefly, the fluorescence intensity increases ∼2–2.5-fold when the protein approaches its vicinity and decreases when the protein moves away (Figure 4A). PIFE displays sharp distance sensitivity under 4 nm range (26,31) and TRBP sliding was visualized by Cy3 signal fluctuations using PIFE (47). In the current configuration, the RNA movement along dsRBP will be monitored because dsRBP is anchored to the surface. For the four dsRBPs, two distinct types of single molecule traces emerged upon binding to RNA: the traces that exhibit robust sliding activity (Figure 4B and Supplementary Figure S4A top) and the ones that show static binding (Figure 4B and Supplementary Figure S4A bottom).

For each dsRBP, we quantified the fraction of sliding molecules out of the total molecules showing dsRBP–dsRNA interaction (Figure 4C). The result shows that TRBP is most likely to slide on dsRNA of both structured and non-structured RNA. On average, 70% of TRBP showed sliding activity on all RNA substrates tested with the exception of tRNA, likely due to the limited length of dsRNA. We note that the observation of TRBP sliding on structured RNA here does not contradict our previous finding where we reported limited TRBP binding to heavily structured RNA that possessed bulky structures including big bulges and extended mismatches along duplex stem (47). The structured RNA used here has minor mismatches and small bulges that do not interfere with TRBP sliding (47). Amongst the other three proteins, only Staufen1 exhibits substantial fraction of sliding molecules on all dsRNAs (20–30%) while ADAR1 and ADAD2 did not show significant level of sliding (Figure 4C, Supplementary Figure S4A). To check if the smPIFE signal fluctuation represents sliding activity, we performed dwell time analysis for TRBP and Staufen1 on three length variants, 25, 40 and 55 bp. The dwell time (δ*t*) reflects the average time it takes for the proteins to complete one round of sliding along dsRNA axis (Supplementary Figure S4B and C). For both TRBP and Staufen1, the dwell time distribution exhibited a clear length dependence, i.e longer time taken for sliding a longer distance, indicating that the PIFE signal fluctuation likely represents sliding activity (Figure 4D and Supplementary Figure S4C). In addition, the average duration of continuous sliding time shows that on average, TRBP spends 30–40 s while Staufen1 spends 10–20 s of time sliding (Figure 4E). We note that these time intervals are likely underestimated due to photobleaching of the fluorescence dye and also the limited data acquisition time of 1 min. Taken together, we show that both TRBP and Staufen1 display sliding activity on dsRNA substrates *in vitro*, albeit to varying degrees.

### Sequence and structure analysis of dsRBD binding to dsRNA

**Figure 5.**
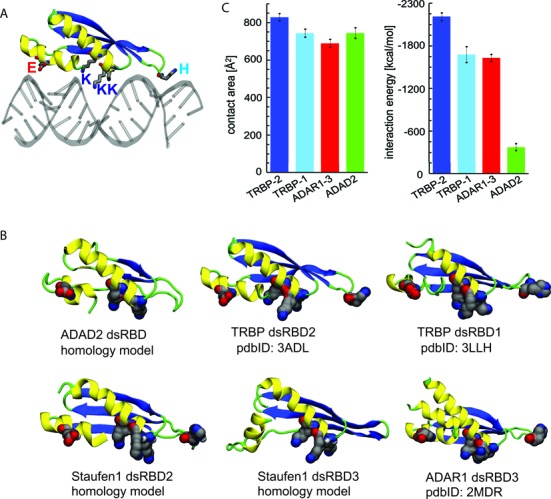
Interactions between dsRNA and individual dsRBDs. (**A**) Canonical binding mode between dsRNA and one dsRBD. The binding mode is shown for TRBP dsRBD2 and dsRNA, based on the crystal structure (pdbID 3ADL). RNA is shown in gray and protein is colored according to secondary structure, where helices are shown in yellow, beta strands in blue, and coils/turns in green. The conserved amino acids (H, E, K) crucial for binding to dsRNA are shown in van der Waals representation, with atoms colored in gray (C), red (O) and blue (N). (**B**) Selected individual dsRBDs of the studied proteins. The structures shown are either crystal structures (labeled with pdbIDs) or homology models (described in ‘Materials and Methods’ section). The coloring scheme for proteins is as in (A). The conserved amino acids crucial for binding to dsRNA are here shown in vdW representation. (**C**) Average contact areas and interaction energies between dsRNA and four selected dsRBDs. The shown values were averaged over the last 35 ns (ADAD2, ADAR1–3) or 85 ns (TRBP dsRBDs) of trajectories, collected after the initial 15 ns-long relaxation.

The type-1 dsRBDs in all four proteins bear high similarity in both amino acid sequence and ‘α_1_-β_1_-β_2_-β_3_-α_2_’ subdomain arrangement (Figure 1B). In particular, the amino acid motifs known to bind to dsRNA are highly conserved across the listed dsRBDs. The three key regions that are critical for interacting with dsRNA are highlighted in the structural models of TRBP–dsRBD2 bound to dsRNA (Figure 5A) and of other dsRBDs bound to dsRNA (Figure 5B). First, there is a conserved E residue (red) in helix α1, which binds to the minor groove of dsRNA. This residue is present in all dsRBDs with the exception of Staufen1 dsRBD3, which nonetheless has a similar Q amino acid in the position. The second conserved dsRNA binding residue is an H residue (light blue) in the loop connecting β1 and β2 strands; this residue is present in all dsRBDs except in ADAD2 dsRBD and Staufen1 dsRBD2. The conserved H residue is known to form a hydrogen bond to the dsRNA minor groove. The third conserved dsRNA binding motif is the KKxxK motif (dark blue) on helix α2; this motif, which binds across dsRNA major groove, is present in all the dsRBDs except in ADAD2 dsRBD (Figure 5B).

To examine the binding strength of individual dsRBDs and dsRNA, we performed MD simulations of several representative dsRBD–dsRNA complexes. Figure 5C shows contact areas and interaction energies between simulated dsRBDs and dsRNA. TRBP-dsRBD2 has the largest contact area and interaction energy, which is in agreement with the highest affinity of TRBP for dsRNA (Figure 2C). TRBP–dsRBD1 and ADAR1–dsRBD3 have reduced affinities for dsRNA. The results presented in Figure 5C are also in agreement with the previously reported result, where TRBP dsRBD2 (*K*_d_ = 113 nM) binds to siRNA with higher affinity than does dsRBD1 (*K*_d_ = 220 nM) (49). In contrast, dsRBD of ADAD2 has a large contact area, but much smaller interaction energy to dsRNA, likely due to multiple hydrophobic residues present close to the dsRNA binding surface, which reduce the magnitude of electrostatic contributions to interaction energy. This minimal binding energy seen in ADAD2 here is consistent with the lowest overall binding affinity observed for ADAD2 shown above (Figure 2C). Our MD simulation result reveals that despite high conservation of RNA binding residues in dsRBDs, the dsRBDs can have a variable affinity for dsRNA. Our data suggests that the calculation of interaction energy may be more useful in predicting the binding affinity of dsRBD and dsRBP than the contact area analysis.

## DISCUSSION

### Protein–RNA interaction examined by single molecule fluorescence

The functions of dsRBPs are implicated in diverse cellular pathways including micro RNA, RNA editing, antiviral signaling and mRNA transport. We sought to profile a set of dsRBPs in three respects. First, we probed the binding specificity of dsRBPs to different lengths and structures of dsRNA that are relevant to cellular RNAs. Second, we examined dynamic properties of dsRBP–dsRNA interactions, based on our previous study of TRBP sliding (47). Third, we investigated the dsRBD–dsRNA interaction strength by performing MD simulations to evaluate contact area and interaction energy. We employed two single molecule assays for this study. The single molecule pull-down assay enabled us to measure the relative affinity of dsRNA substrates toward dsRBP. This platform provides several main advantages over other methods such as electrophoretic mobility shift assay (EMSA). The proteins can be directly pulled down from cell lysate without being processed through purification steps, hence preserving the native context of the protein in cells. Second, it enables one to detect not only stable binding but also weak or transient binding events, which is not possible with gel-based assays. Third, it allows observation of single dsRBP-dsRNA interaction. In addition, the same platform was used for performing single molecule PIFE assay to detect the dynamic sliding of dsRNA on dsRBP visualized by signal fluctuation of a single fluorophore attached to RNA. Here, the smPIFE assay provides a unique opportunity to detect such dynamic motion because FRET measurement requires fluorescent labeling of the protein, which cannot be done in this scheme. Furthermore, it is advantageous to work with unlabeled protein to avoid potential disruption by the fluorescent dye that may perturb the protein activity.

### dsRBPs’ binding affinity to dsRNAs varies despite highly conserved dsRBD

There are ∼30 known dsRBD-containing proteins in human cells. Most of the dsRBDs, including dsRBD1 and dsRBD2 of TRBP, dsRBD2 of Staufen1 and dsRBD3 of ADAR1, possess the canonical dsRNA binding motifs that contact the minor–major–minor pattern on A-form dsRNA helix (Figure 5A). One exception is ADAD2 dsRBD, which is missing one K residue from the KKxxK motif and the H residue on the loop connecting β1 and β2 strands. The loop contains only hydrophobic residues, making it unlikely to form hydrogen bond contact with the minor groove of dsRNA. The lack of K and H amino acids make ADAD2 dsRBD interactions with dsRNA substantially weaker than for other dsRBDs (Figure 5C). In agreement, we obtained the lowest overall binding affinity of ADAD2 to all types of RNAs tested (Figure 3C). On the other hand, dsRBD3 of ADAR1 has all the consensus dsRBD residues, yet ADAR1 exhibits weak binding to most RNAs tested. Taken together, the structural model analysis (Figure 4A) and relative affinity results (Figure 2C) suggests that the binding affinity of dsRBP to dsRNA may be correlated to the predicted binding affinity of single dsRBDs in some cases, but not in all cases.

Our binding affinity results indicate a plausible distinction between the strong (TRBP) and weak binders (all others) to dsRNA, although most of the dsRBDs contain all the dsRNA binding consensus residues (besides ADAD2 and Staufen1-dsRBD3). A possible reason for this discrepancy in RNA binding strength might be the presence of a basic residue adjacent to the KKxxK motif (either K or R) observed in several dsRBDs; MD simulations show that such R residue of TRBP dsRBD2, which is not present in dsRBD1, enhances the binding strength of only dsRBD2 to dsRNA (50). Alternatively, other structural features in ADAD2, ADAR1 and Staufen1 proteins can modulate their dsRNA binding and dynamic properties; for example, dsRBDs could be sterically occluded from interacting with RNA, have competitive binding to other protein domains, or require dimerization for high-affinity binding to dsRNA. In fact, ADAR1 is known to bind to 19-bp siRNA with high affinity in the dimer form (*K*_d_ = 0.21 nM), or with lower affinity when dimerization is prevented due to a single mutation (*K*_d_ = 2.2 nM) (51).

### The number of dsRBDs in dsRBPs may not correlate with the RNA binding affinity

It is interesting and puzzling that the number of type-1 dsRBD per protein is highly variable in the dsRBP family, ranging from one to five. It is not known if the number of dsRBDs determines its binding strength to dsRNA or if they contribute to substrate specificity of the dsRBP. We characterized four dsRBPs with a different number of dsRBDs. Our data shows that the binding affinity of dsRBPs toward RNA substrates is not necessarily correlated with the number of dsRBD-Is (Figure 2C). ADAR1 with the highest number (3) of dsRBD-Is did not show the highest affinity whereas TRBP with only two dsRBD-Is exhibited the strongest affinity to all substrates. Our data also indicates that the number of dsRBDs may not contribute to binding preference to structured versus unstructured dsRNA or to length of dsRNA. For instance, ADAR1 with three dsRBDs showed the least variance in binding affinity toward studied RNA substrates, while Staufen1 with two dsRBDs showed preferred binding to structured RNA (Figure 3D). We conclude that multiple dsRBDs may not be responsible for discriminating dsRNA length or structure on their own. We note that our conclusion is based on the subset of structural variants chosen for this study and may not reflect a situation in cells.

Based on the experimentally determined *K*_d_ values for dsRNA binding to several TRBP and ADAR1 constructs (49,51), we can further examine the effect of multiple dsRBDs on protein–dsRNA binding strength (Supplementary Figure S5A). The *K*_d_ values of TRBP–dsRBD2 and TRBP–dsRBD1 are 113 and 220 nM (49), respectively, indicating that TRBP–dsRBD2 has stronger binding to dsRNA. These *K*_d_ values can be used to estimate a lower bound on the *K*_d_ value of the TRBP–RBD1+2 construct, in which TRBP–RBD1 and TRBP–RBD2 are linked by a long flexible linker of 61 amino acids. If we assume that the dsRBDs act independently when flexibly bound, we can predict the *K*_d_ by obtaining a product of *K*_d_ values of individual dsRBDs (Supplementary Figure S5B). However, the experimental *K*_d_ value of TRBP–RBD1+2 (250 pM) is an order of magnitude higher than the theoretically estimated lower-bound *K*_d_ (24.9 pM), which indicates that dsRBDs are not completely independent of each other in TRBP. Therefore, the presence of multiple dsRBDs in a protein is likely to strengthen protein–dsRNA binding and lower the *K*_d_ value, but not to the strongest possible binding associated with the predicted lower-bound*K*_d_ value.

### Dynamic sliding may be due to flexible linkers between dsRBDs

The interaction between dsRBP and RNA probed by the smPIFE assay revealed two distinct binding modes, sliding and static (Figure 4B). Based on the sequence comparison of the dsRBPs, we hypothesize that sliding can be enhanced by flexibly linked dsRBDs. TRBP, which displays the most sliding, has two dsRBDs connected by a long flexible 61 amino acids (aa) linker. In comparison, Staufen1 contains two type-1 dsRBDs connected by a short 25aa linker, and it has a much lower fraction of protein molecules sliding along RNA substrates. Another dsRBP, PACT, has a domain composition similar to TRBP, but its two type-1 dsRBDs are connected with a short 25aa linker. Similar to Staufen1, PACT showed a substantially lower propensity to slide on dsRNA than does TRBP (unpublished data).

Along the same line, we expect that rigid or protein-embedded dsRBDs may not slide well on RNA substrates. In the case of ADAD2, its dsRBD has a large content of hydrophobic residues on its surface, making it likely to be protected by other proteins or domains from water exposure. Consequently, the only dsRBD in ADAD2 could be less flexible and occluded from optimal dsRNA contact by other domains or partner proteins. ADAR1 is significantly larger than the other proteins studied here, including several additional domains, like Z-DNA binding and deaminase domains. It is plausible that such bigger and complex domain arrangement leads to a more rigid structure and, hence, restricts the dynamic behavior of the dsRBDs on RNA. Since both ADAD2 and ADAR1 are RNA deaminases (17), they need to translocate to the A to I editing site. The low fraction of sliding molecules for both of them suggests that they may collaborate with other protein partners, like other mobile dsRBPs or motor proteins. In agreement, previous work identified ILF3, which contains two dsRBD-Is with about 60aa linker space in between, as a partner protein of ADAR1 in a dsRNA-binding-dependent manner (52).

Our study offers a new approach to investigate RNA–protein interaction that may lead to more quantitative and deeper understanding of molecular mechanism.

## Supplementary Material

SUPPLEMENTARY DATA
