# Supplementary material for: Dynamic profiling of double-stranded RNA binding proteins
Source: Nucleic Acids Res. 2015 Jul 16;43(15):7566–76. doi: 10.1093/nar/gkv726 (PMC4551942; doi:10.1093/nar/gkv726)
Supplement: SUPPLEMENTARY DATA [file supp_43_15_7566__index.html]

Dynamic profiling of double-stranded RNA binding proteins — SUPPLEMENTARY DATA 

# Dynamic profiling of double-stranded RNA binding proteins

## SUPPLEMENTARY DATA

- SUPPLEMENTARY DATA
