# Supplementary material for: Dynamic profiling of double-stranded RNA binding proteins
Source: Nucleic Acids Res. 2015 Jul 16;43(15):7566–76. doi: 10.1093/nar/gkv726 (PMC4551942; doi:10.1093/nar/gkv726)
Supplement: SUPPLEMENTARY DATA [file supp_gkv726_nar-01592-y-2015-File007.docx]

Supplementary Information

**Dynamic profiling of double-stranded RNA binding proteins**

Xinlei Wang^1,2,3^, Lela Vukovic^2,4^, Hye Ran Koh^2,3,4^ Klaus Schulten^2,4,5^, Sua Myong^1,2,3,5^

^1^Department of Bioengineering, University of Illinois at Urbana-Champaign, Urbana, Illinois 61801, United State

^2^Center for the Physics of Living Cells, University of Illinois at Urbana-Champaign, Urbana, Illinois 61801, United States

^3^Institute for Genomic Biology, University of Illinois, 1206 W. Gregory St. Urbana IL 61801, USA

^4^Department of Physics, University of Illinois at Urbana-Champaign, Urbana, Illinois 61801, United States

^5^Biophysics and Computational Biology, University of Illinois, 1110 W. Green St. Urbana, Illinois 61801

Correspondence should be addressed to S.M. (smyong@illinois.edu)

Supplementary Figure 1

Supplementary Figure 2

Supplementary Figure 3

Supplementary Figure 4

Supplementary Figure 5

Supplementary Table 1

**Supplementary Figure S1**


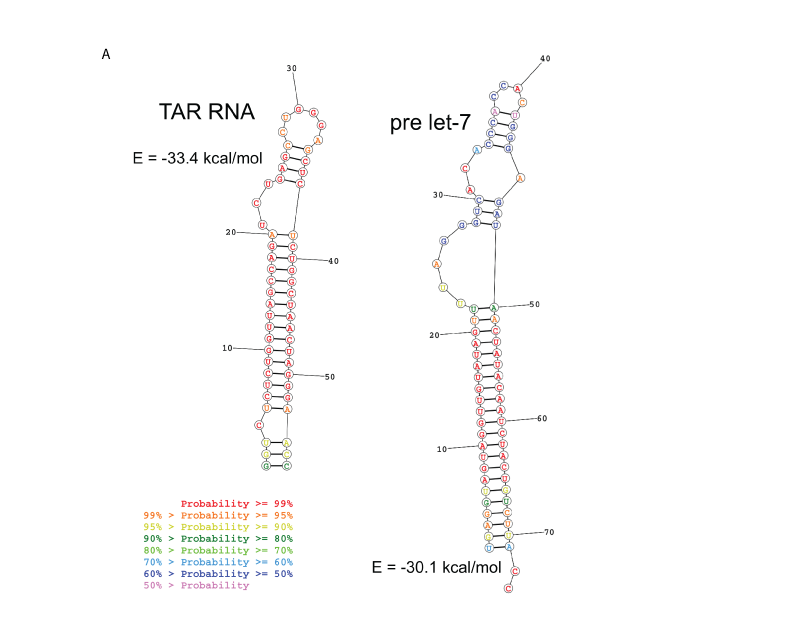


**Figure S1. Predicted structure of TAR RNA and pre let-7. A)** TAR RNA and pre let-7 secondary structure predictions with the lowest free energies, obtained with the RNAstructure webserver [^6^](#_ENREF_6). 3D structures of TAR RNA and prelet-7, shown in Figure 2B, are based on the predictions shown here. 3D structures were obtained through the 3dRNA web server [^7^](#_ENREF_7).

Supplementary Figure S2


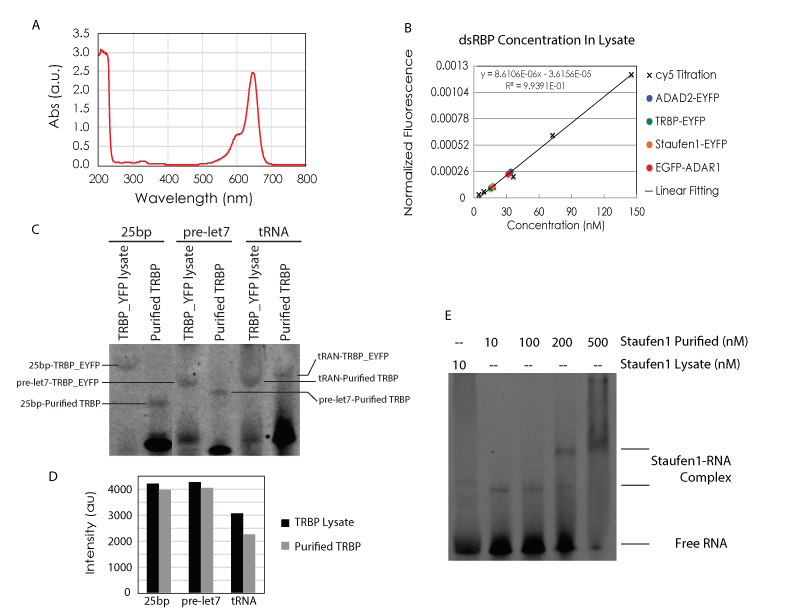


**Figure S2. Characterization of protein concentration, purity and dimer/monomer status of dsRBPs from lysate. A)** Spectrophotometry absorption measurement of free Cy5 dye sample. **B)** Determination of dsRBP concentration based on the calibration curve generated from Cy5 dye. Cy5 dye sample was serially diluted to generate a standard fluorescence-to-concentration line. Plotting normalized fluorescence intensity of four dsRBPs from lysate onto the calibration line enables estimation of concentration for each dsRBPs in lysate. **C)** EMSA assay to test the affinity of TRBP_YFP from lysate vs. purified TRBP to three representative RNA substrates labeled with Cy3. **D)** Quantification of shifted protein-RNA complex bands in C). **E)** EMSA assay to test concentration dependent oligomerization of purified Staufen1 interacting with Cy3-tRNA in parallel with Staufen1 lysate.

Supplementary Figure S3


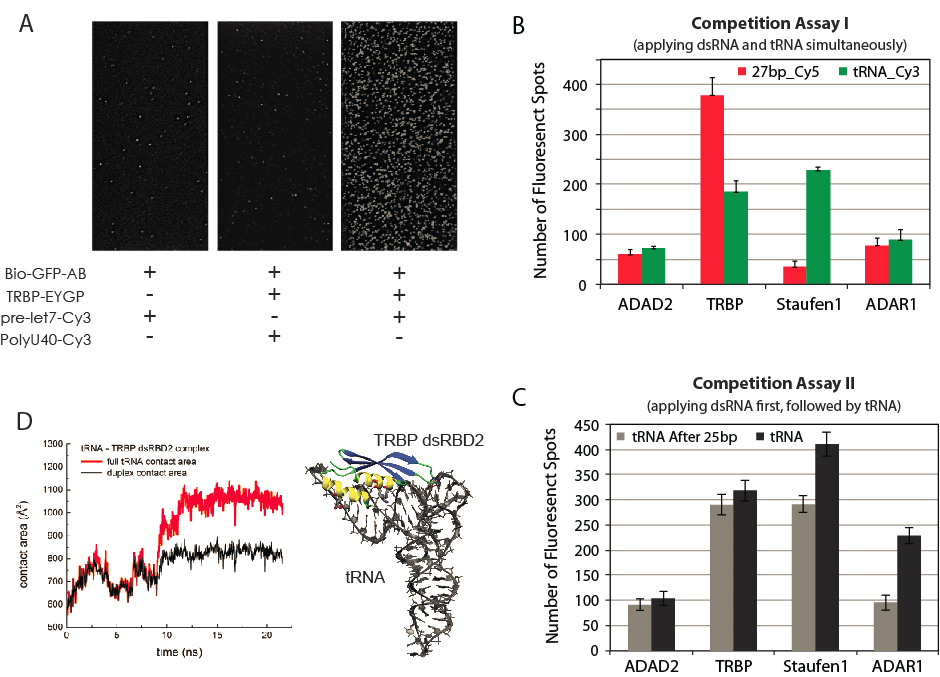


**Figure S3. Nonspecific binding test and competitive binding affinity of dsRBPs towards tRNS vs. dsRNA A)** Testing nonspecific binding by omitting cell lysate, pre-let7 RNA and adding U40 ssRNA. **B)** Competitive binding assay between duplex Cy3-tRNA and Cy5-dsRNA (27 bp) on single molecule platform by applying the same concentration (500pM each) of the two RNAs at each of the four dsRBPs immobilized surface. **C)** Competitive binding assay in which dsRNA pre-bound to dsRBP was competed away by labeled tRNA (gray bar). The tRNA binding result is put in as a comparison (black bar). The tRNA was able to compete against dsRNA to sufficient degree for all four proteins tested. **D)** MD simulation of dsRBD2 of TRBP to binding to dsRNA vs. tRNA. The interaction to tRNA is comparable to dsRNA.

**Supplementary Figure S4**

**
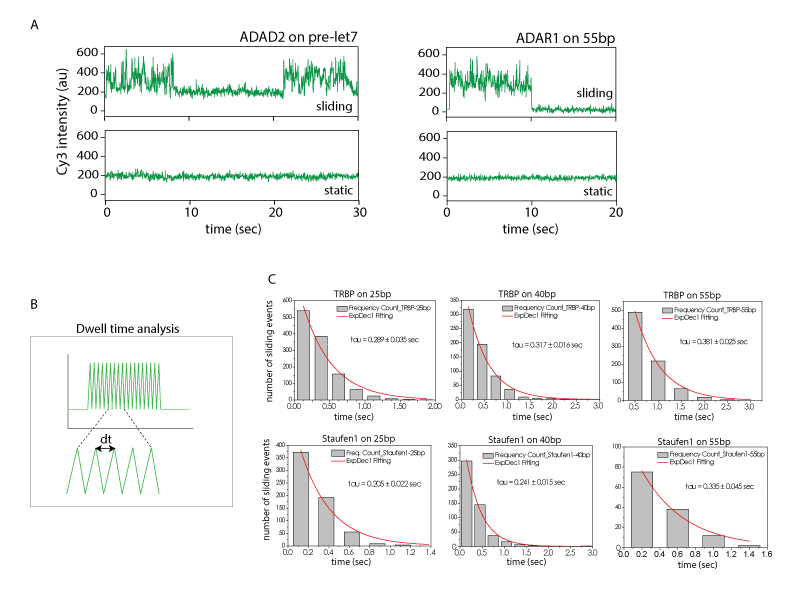
**

**Figure S4 Dwell time analysis of TRBP and Staufen1 on length variant dsRNA A)** Two different types (sliding vs. static) of smPIFE traces observed in ADAD2-pre-let7 and ADAR1-55bp dsRNA. **B)** Schematic of dwell time (δt) collection from smPIFE data. The time intervals between PIFE peak to peak were collected from over 500 sliding events for dwell time analysis. **C)** Dwell time distribution of TRBP and Staufen1 sliding on 25, 40 and 55 bp of dsRNA.

Supplementary Figure S5


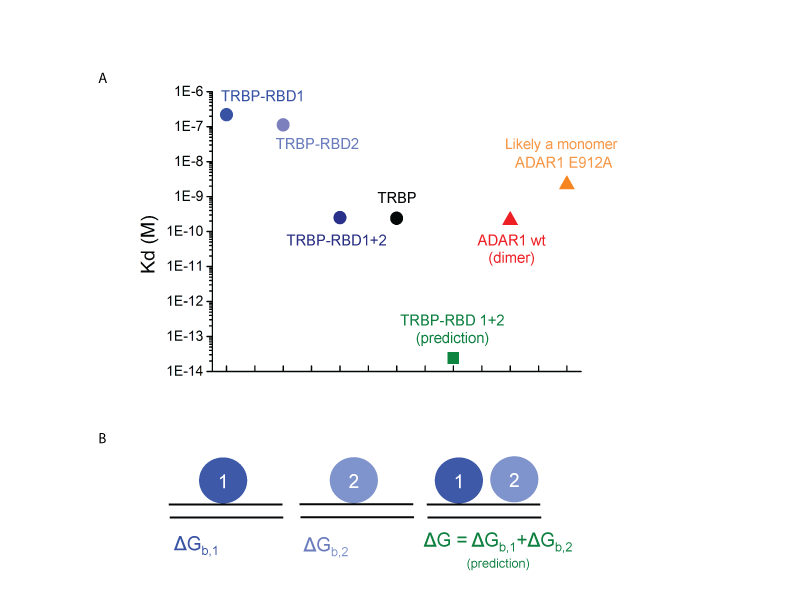


**Figure S5. Dissociation constants (K_d_) of dsRNA and several dsRBPs or their parts. A)** K_d_ values of several individual dsRBDs (TRBP-RBD1, TRBP-RBD2 [^15^](#_ENREF_15)), dsRBD constructs (TRBP-RBD1+2 [^15^](#_ENREF_15)), and dsRBPs (TRBP [^15^](#_ENREF_15), ADAR1 [^16^](#_ENREF_16), ADAR1 E912A [^16^](#_ENREF_16); K_d_ values for ADAR1 are reported for siRNA substrate. The predicted K_d_ value for dsRNA and TRBP-RBD1+2 construct, based on the assumption that dsRBDs in this construct act independently, is also shown in green. **B)** A scheme showing the relationship between binding free energies of two TRBP dsRBDs binding independently to dsRNA.

**Supplementary Table 1**

| **Name** | **Sequence** |
| --- | --- |
| 25bp RNA | 5’- rGrCrUrUrGrUrCrGrGrGrArGrCrGrCrCrArCrCrCrUrCrUrGrC-3’ (up) |
|  | 5’- rGrCrArGrArGrGrGrUrGrGrCrGrCrUrCrCrCrGrArCrArArGrC-DY547-3’ (down) |
|  |  |
| 40bp RNA | 5’- rGrCdTrUrArArCrArArCrCrArGrArUrCrArArArGrArArArArArArCrArGrArCrArUrU  rGrUrCrA-3’ (up) |
|  | 5’- rUrGrArCrArArUrGrUrCrUrGrUrUrUrUrUrUrCrUrUrUrGrArUrCrUrGrGrUrUrGrU  rUrArArGrCrGrU-DY547-3’ (down) |
|  |  |
| 55bp RNA | 5’- rArCrGrCrUrUrArArCrArArCrCrArGrArUrCrArArArGrArArArArArArCrArGrArCrA  rUrUrGrUrCrArArUrUrGrCrArArArGrCrArArArArA-3’ (up) |
|  | 5’- rUrUrUrUrUrGrCrUrUrUrGrCrArArUrUrGrArCrArArUrGrUrCrUrGrUrUrUrUrUrU  rCrUrUrUrGrArUrCrUrGrGrUrUrGrUrUrArArGrCrGrU-DY547-3’ (down) |
|  |  |
| Pre-let7 | 5’-cy3-rUrGrArGrGrUrArGrUrArGrGrUrUrGrUrArUrArGrUrUrUrUrArGrGrGrUrCrArCrA  rCrCrCrArCrCrArCrUrGrGrGrArGrArUrArArCrUrArUrArCrArArUrCrUrArCrUrGrU  rCrUrUrArCrC-3’ |
|  |  |
| TAR | 5’-cy3-rGrGrUrCrUrCrUrCrUrGrGrUrUrArGrCrCrArGrArUrCrUrGrArGrCrCrUrGrGrGrA  rGrCrUrCrUrCrUrGrGrCrUrArArCrUrArGrGrGrArArCrC-3’ |
|  |  |
| tRNA | 5’-cy3- rGrGrGrArArGrCrCrCrGrGrArUrArGrCrUrCrArGrUrCrGrGrUrArGrArGrCrArUrC  rArGrArCrUrUrUrUrArArUrCrUrGrArGrGrGrUrCrCrArGrGrGrUrUrCrArArGrUrCrC  rCrUrGrUrUrCrGrGrGrCrGrCrCrA-3’ |
|  |  |
| PolyU40 | 5’ –rUrUrUrUrUrUrUrUrUrUrUrUrUrUrUrUrUrUrUrUrUrUrUrUrUrUrUrUrUrUrUrUrUrUrUrUrUrUrUrU-cy3-3’ |

REFERENCE

(1) Roy, R.; Hohng, S.; Ha, T. *Nature methods* **2008**, *5*, 507.

(2) Jain, A.; Liu, R.; Xiang, Y. K.; Ha, T. *Nature Protocols* **2012**, *7*, 445.

(3) Hwang, H.; Kim, H.; Myong, S. *Proc Natl Acad Sci U S A* **2011**, *108*, 7414.

(4) Hwang, H.; Myong, S. *Chemical Society reviews* **2014**, *43*, 1221.

(5) Lu, X. J.; Olson, W. K. *Nucleic Acids Research* **2003**, *31*, 5108.

(6) Reuter, J. S.; Mathews, D. H. *BMC Bioinformatics* **2010**, *11*.

(7) Zhao, Y.; Huang, Y.; Gong, Z.; Wang, Y.; Man, J.; Xiao, Y. *Scientific Reports* **2012**, *2*.

(8) Haas, J.; Roth, S.; Arnold, K.; Kiefer, F.; Schmidt, T.; Bordoli, L.; Schwede, T. *Database* **2013**, *2013*.

(9) Han, W.; Wan, C.-K.; Jiang, F.; Wu, Y.-D. *Journal of Chemical Theory and Computation* **2010**, *6*, 3373.

(10) Han, W.; Schulten, K. *Journal of Chemical Theory and Computation* **2012**, *8*, 4413.

(11) Phillips, J. C.; Braun, R.; Wang, W.; Gumbart, J.; Tajkhorshid, E.; Villa, E.; Chipot, C.; Skeel, R. D.; Kalé, L.; Schulten, K. *Journal of Computational Chemistry* **2005**, *26*, 1781.

(12) Hornak, V.; Abel, R.; Okur, A.; Strockbine, B.; Roitberg, A.; Simmerling, C. *Proteins: Structure, Function and Genetics* **2006**, *65*, 712.

(13) Pérez, A.; Marchán, I.; Svozil, D.; Sponer, J.; Cheatham Iii, T. E.; Laughton, C. A.; Orozco, M. *Biophysical Journal* **2007**, *92*, 3817.

(14) Darden, T.; York, D.; Pedersen, L. *The Journal of Chemical Physics* **1993**, *98*, 10089.

(15) Yamashita, S.; Nagata, T.; Kawazoe, M.; Takemoto, C.; Kigawa, T.; Güntert, P.; Kobayashi, N.; Terada, T.; Shirouzu, M.; Wakiyama, M.; Muto, Y.; Yokoyama, S. *Protein Science* **2011**, *20*, 118.

(16) Yang, W.; Wang, Q.; Howell, K. L.; Lee, J. T.; Cho, D. S. C.; Murray, J. M.; Nishikura, K. *Journal of Biological Chemistry* **2005**, *280*, 3946.
